# The genome sequence of *Tadarida brasiliensis* I. Geoffroy Saint-Hilaire, 1824 [Molossidae; Tadarida]

**DOI:** 10.12688/wellcomeopenres.20603.1

**Published:** 2024-02-29

**Authors:** Cara F. Webster, Michael Smotherman, Martin Pippel, Thomas Brown, Sylke Winkler, Myrtani Pieri, Meike Mai, Eugene W. Myers, Emma C. Teeling, Sonja C. Vernes, The Bat1K Consortium

**Affiliations:** 1 Department of Biology, Texas A&M University, College Station, Texas, USA; 2 Max Planck Institute of Molecular Cell Biology and Genetics, Dresden, Pfotenhauerstr. 108, 01307 Dresden, Germany; 3 Center for Systems Biology, Dresden, Pfotenhauerstr. 108, 01307 Dresden, Germany; 4 DRESDEN concept Genome Center, Center for Molecular and Cellular Bioengineering, Technische Universitat, Dresden, 01307 Dresden, Germany; 5 Department of Life Sciences, School of Life and Health Sciences, University of Nicosia, Nicosia, Cyprus; 6 School of Biology, University of St Andrews, St Andrews, UK; 7 School of Biology and Environmental Science, University College Dublin, Dublin, Ireland; 8 Wellcome Sanger Institute, Wellcome Genome Campus, Cambridgeshire, CB10 1SA, UK; 9 Neurogenetics of Vocal Communication Group, Max Planck Institute for Psycholinguistics, Nijmegen, The Netherlands

**Keywords:** Tadarida brasiliensis, genome sequence, chromosomal, Bat1K

## Abstract

We present a genome assembly from an individual male *Tadarida brasiliensis* (The Brazilian free-tailed bat; Chordata; Mammalia; Chiroptera; Molossidae). The genome sequence is 2.28 Gb in span. The majority of the assembly is scaffolded into 25 chromosomal pseudomolecules, with the X and Y sex chromosomes assembled.

## Species taxonomy

Eukaryota; Metazoa; Eumetazoa; Bilateria; Deuterostomia; Chordata; Craniata; Vertebrata; Gnathostomata; Teleostomi; Euteleostomi; Sarcopterygii; Dipnotetrapodomorpha; Tetrapoda; Amniota; Mammalia; Theria; Eutheria; Boreoeutheria; Laurasiatheria; Chiroptera; Yangochiroptera; Molossidae; Tadarida; *Tadarida brasiliensis,* (I. Geoffroy Saint-Hilaire, 1824) (NCBI:txid9438; subordinal taxonomy updated per Teeling *et al*, ( Teeling *et al.*, 2005).

## Introduction

**Figure 1.**
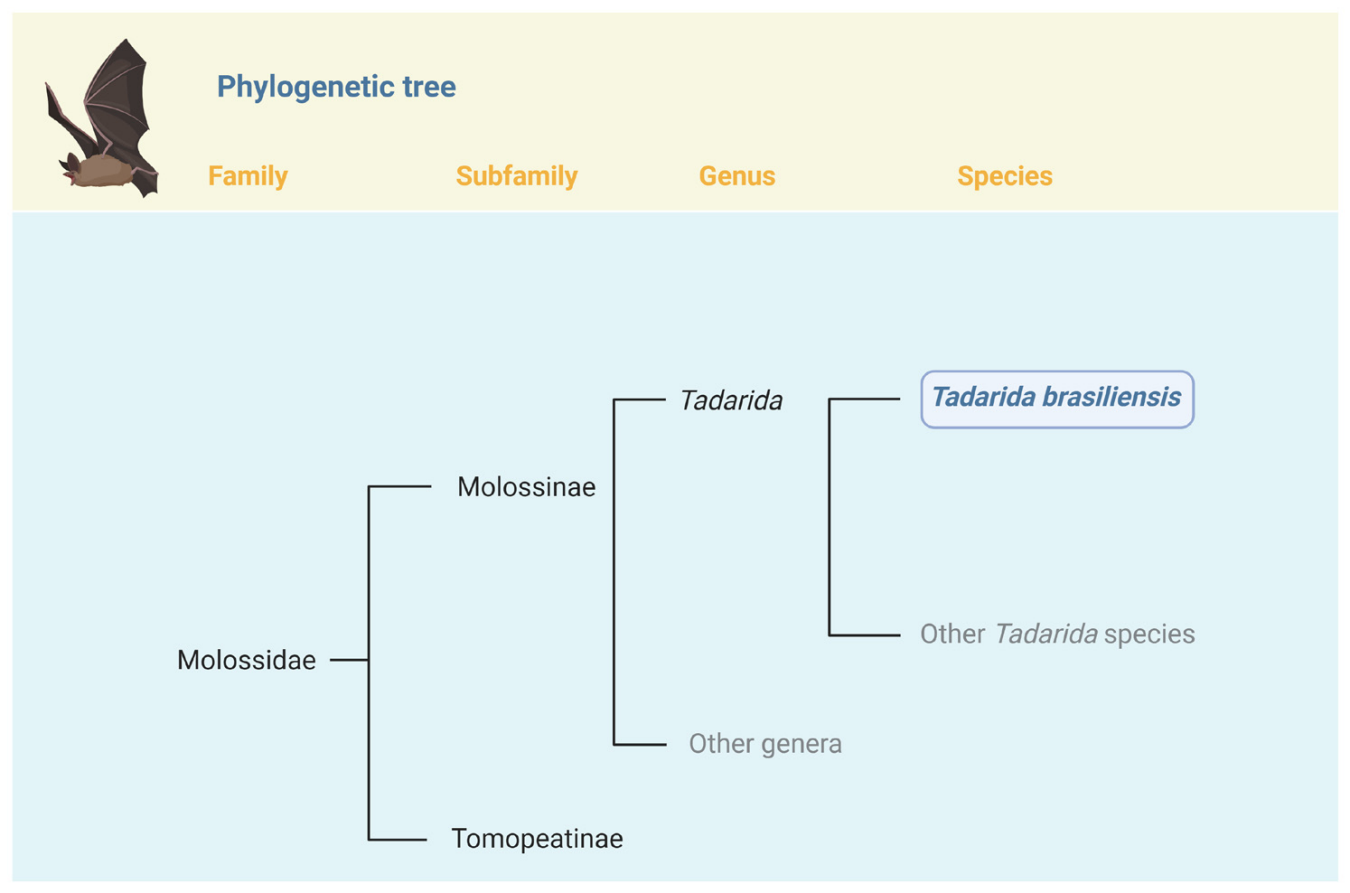
Position of *Tadarida brasiliensis* in the phylogeny of Family *Molossidae*. *Tadarida brasiliensis* is one of 8 species currently recognized in the genus *Tadarida* (Rafinesque, 1814). *Tadarida* belongs to the subfamily Molossinae ( Gervais, 1856), which currently includes 20 genera and 132 species. FIgure created with BioRender.com.

*Tadarida brasiliensis*, commonly known as the Mexican free-tailed bat or the Brazilian free-tailed bat, is medium-sized New World insectivorous bat. Belonging to the family *Molossidae, Tadarida* is one of 21 genera that comprise the 4 ^th^ largest family in *Chiroptera ( Simmons, 2023).* The genus of *Tadarida* contains 8 species, with *T. brasiliensis* being the only New World bat of the genus ( Figure 1). While some genera with *Molossidae* show support for monophyly, *Tadarida* does not show evidence for forming a monophyletic clade ( Agnarsson *et al.*, 2011). Since *T. brasiliensis* is the only New World bat of this genus, a subgenus classification of *Rhizomops* has been previously proposed ( Legendre, 1984), but morphological and genetic evidence does not support this distinction ( Gregorin & Cirranello, 2016; Ammerman *et al.*, 2012). Genetic evidence from four genes produces a clade specifically formed of *T. brasiliensis*, *Tadarida aegyptiaca* and *Sauromys petrophilus ( Ammerman *et al.*, 2012)*, but more recent analysis using morphological evidence does not support this clade ( Gregorin & Cirranello, 2016). The closest relative of *T. brasiliensis* is *T. aegyptiaca*, with *T. brasiliensis* showing a higher relation to Old World molossids with the last shared common ancestor with the New World clade being 29 mya ( Ammerman *et al.*, 2012).

*T. brasiliensis* is one of the most widely distributed mammals in the New World, and one of the most abundant bat species. According to the International Union for Conservation of Nature (IUCN), *T. brasiliensis* is listed as Least Concern ( Barquez *et al.*, 2015). The geographic range of the species includes most of the United States, Mexico, Central America, and southwestern South America, including Greater and Lesser Antilles ( Wilkins, 1989). Due to the species’ large range, and proposed behavioral and morphological differences, *T. brasiliensis* was once thought to comprise up to nine subspecies ( Schwartz, 1955). However, the population structure from genetic studies and morphological evidence do not confirm any subspecies classification and support gene flow between mainland and island populations ( Morales *et al.*, 2016; Morales *et al.*, 2018). Instead, phenotypic differences are hypothesized to be correlated to climatic variation across regions and individuals are recommended to be categorized as migratory or non-migratory ( Morales *et al.*, 2016). The large geographic range is most likely related to the species’ large dispersal capabilities.

**Figure 2.**
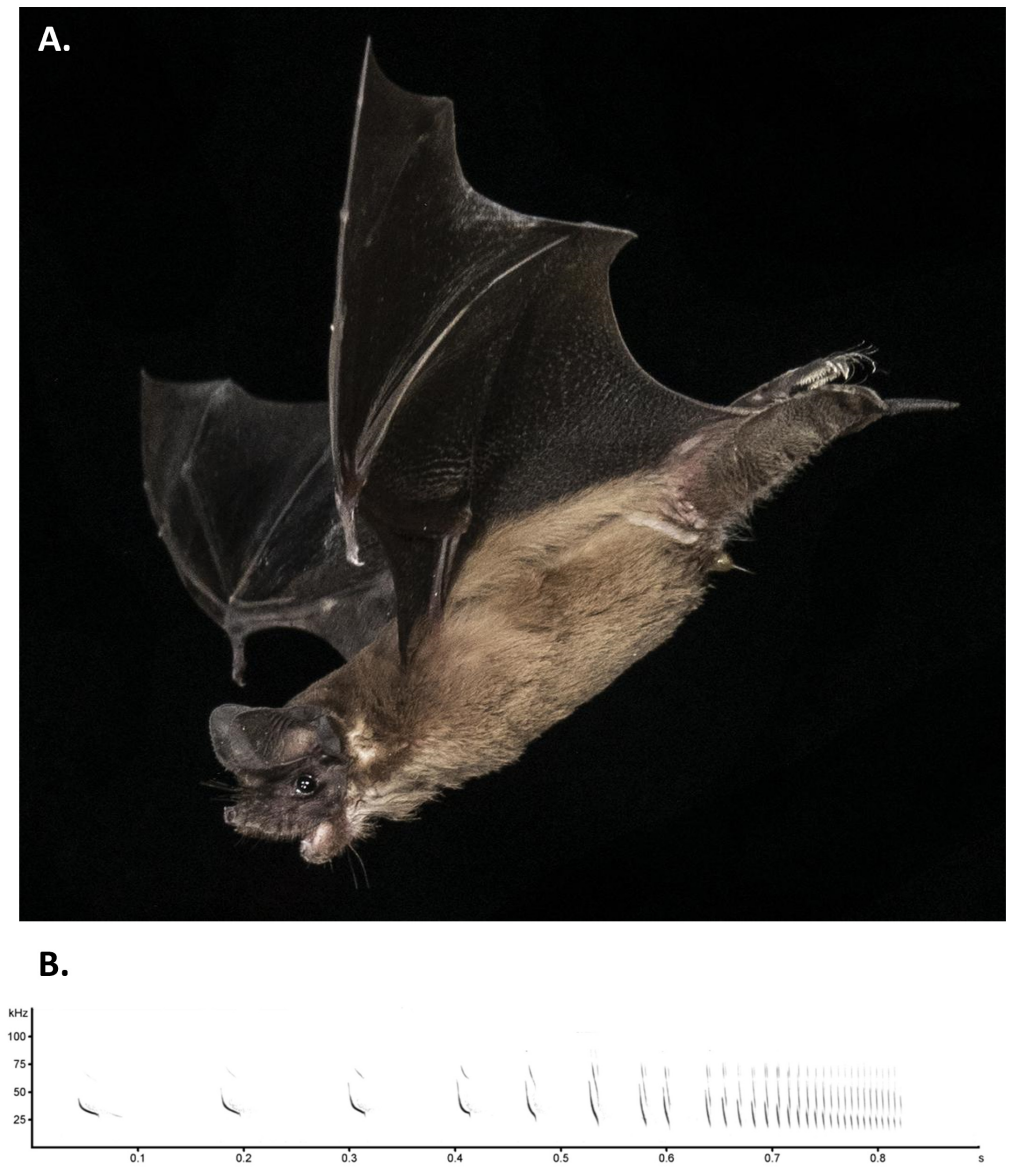
*Tadarida brasiliensis* **A**) Adult individual of the Mexican free-tailed bat, *Tadarida brasiliensis.* Note the tail extending beyond the uropatagium, short wrinkled snout, and hairs extending beyond toes. Photo courtesy of Brock and Sherri Fenton with permission, Windsor Cave, Jamaica. **B**) An echolocation pulse sequence emitted by *Tadarida brasiliensis* while foraging over a pond. This sequence begins with a typically shallow frequency-modulated search-phase pulse and ends with a terminal buzz.

One of the defining traits of *T. brasiliensis* is the “free tail”, where the tail extends beyond the uropatagium which is characteristic of the *Molossidae* family ( Figure 2A). *T. brasiliensis* is the smallest of the New World molossids with an adult weight range of 11–14 g, average total body length of 95 mm, and the average forearm length of 42 mm ( Schmidly, 2018). The species has a short velvety brown pelage, with long hairs on the feet that extend past the toes ( Wilkins, 1989). The snout is short with wrinkled lips. The ears, when pressed forward, do not extend past the snout and do not meet at the midline. The species is not strongly sexually dimorphic, but reproductively active males have an enlarged gular gland which is a sebaceous gland found on the suprasternal neck region ( Krutzsch *et al.*, 2002). This gland shows seasonal functionality and during the breeding season secretes a thick, oily, odorous substance in reproductive adult males.

*T. brasiliensis* is known for forming large colonies, with a single colony sometimes containing several million individuals. The densest populations of the species are found in Texas, where in the summer an estimated 95–104 million bats (primarily females forming maternity colonies) occupy a few select caves known as guano caves ( Schmidly, 2018). *T. brasiliensis* primarily roost in caves or man-made structures such as buildings or under bridges but have also been found in hollow trees in the southeastern US ( Wilkins, 1989).

This species is classified as a migratory bat with some of the longest recorded bat migrations. *T. brasiliensis* is estimated to have an annual migration as far as 1500 km ( Villa-R & Cockrum, 1962), traveling from central and southwestern United States southward into Mexico ( Cockrum, 1969). While shorter seasonal movements in temperate bats are not uncommon, mostly in response to unfavorable climate ( Popa-Lisseanu & Voigt, 2009), longer migrations as seen in *T. brasiliensis* are rarer in bats. While temperature changes could be triggering this movement, another potential reason for this annual trip is the seasonal availability and distribution of food resources, such as movements of migratory moths ( Russell *et al.*, 2005). Even during nightly foraging trips, individuals may travel more than 160 km from their roost to a foraging site and back in one evening, maintaining horizontal flight speeds of up to 44 m/s ( Davis *et al.*, 1962; McCracken *et al.*, 2016). This high dispersal capability may be responsible for the lack of distinct population structure across the wide species range. However, there are differences in migratory behavior based on geographic location and sex. Previous banding experiments propose sedentary or non-migratory subpopulations in parts of the United States ( Cockrum, 1969), but no genetic differentiation has been found to account for this difference in migratory phenotype ( Russell *et al.*, 2005). Also, females seem to travel further with more males forming resident populations in the winter or not traveling as far into Mexico ( Russell *et al.*, 2005).

*T. brasiliensis* is an aerial insectivore that relies upon echolocation to navigate and forage for prey ( Figure 2B). In open spaces this species emits a shallow frequency-modulated echolocation pulse that descends from approximately 25 to 20 kHz over a 15–20 ms duration. When approaching obstacles or prey, pulse durations are progressively shortened to 2 ms while the starting frequency of the fundamental harmonic is concomitantly raised to approximately 50 kHz, with higher (2 ^nd^ and 3 ^rd^) non-overlapping harmonics also becoming prominent in the signal ( Schwartz *et al.*, 2007; Simmons *et al.*, 1978). *T. brasiliensis* is known to forage at high altitudes, with recorded foraging activity on migratory noctuid moths ( *Lepidoptera*) occurring as high as 3000 m ( Williams *et al.*, 1973). However, *T. brasiliensis* can also forage near ground and their diverse diet varies seasonally depending on species abundance and availability ( McCracken *et al.*, 2021; Ross, 1961).

Previously, there has only been a short read genome assembly of *T. brasiliensis* available (GenBank accession: GCA_004025005.1) which was generated as a part of the Zoonomia Project ( Zoonomia, 2020). Notable sequencing projects utilizing this assembly consist of comparative genomics analyses of diet, visual system adaptations based on foraging style, immunity and metabolic adaptations, and longevity across several bat species ( Blumer *et al.* 2022; Davies *et al.*, 2020; Fushan *et al.*, 2015; Moreno Santillan *et al.*, 2021; Potter *et al.*, 2021). As for future applications with the new reference quality long read genome assembly reported herein, *T. brasiliensis* has previously been proposed as an ideal mammal model for the genetic and epigenetic basis of migration, mostly due to the widespread species range, abundant population, and variation of the phenotype ( Merlin & Liedvogel, 2019). Functional genomics projects should be fruitful in this species compared to other North American bats due to their high population levels and therefore less conservation pressure, as sample sizes are often a limiting factor. Although there is continued potential for comparative projects across multiple bat species, access to this genome assembly will hopefully encourage more work regarding the genetic basis for *T. brasiliensis*’ unique traits.

## Genome sequence report

The genome was sequenced from a single male *Tadarida brasiliensis* collected from the Texas A&M campus of College Station, Brazos County, Texas, USA. A total of 39-fold coverage in Pacific Biosciences Hi-Fi long reads (contig N50 86 Mb) was generated after removal of all reads shorter than 10kb. Primary assembly contigs were scaffolded with chromosome confirmation Hi-C data. The final assembly has a total length of 2.28 Gb in 147 sequence scaffolds with a scaffold N50 of 111 Mb ( Table 1). The majority, 98.44%, of the assembly sequence was assigned to 25 chromosomal-level scaffolds, representing 23 autosomes (numbered by sequence length, and the X and Y sex chromosomes). Chromosomal pseudomolecules in the genome assembly of *Tadarida brasiliensis* are shown in Table 2. The assembly has a BUSCO ( Simao *et al.*, 2015) completeness of 96.3% using the laurasiatheria reference set. While not fully phased, the assembly deposited is of one haplotype.

**Table 1.** Genome data for *Tadarida brasiliensis*.

*Project accession data*	
Assembly identifier	DD_mTadBra1_pri
Species	*Tadarida brasiliensis*
Specimen	mTadbra1
NCBI taxonomy ID	txid9438
BioProject	Bat1K: Accession: PRJNA489245; ID: 489245 Tadarida: Accession: PRJNA972445ID: 972445
BioSample ID	SAMN35075070
Isolate information	Male - muscle
*Raw data accessions*	
Pacific Biosciences SEQUEL II	SRX20532109
Hi-C Illumina	SRX20532110, SRX20532111
10X linked-reads	SRX20532112
Assembly accession	GCA_030848825.1
Accession of Alternative haplotype	GCA_030848815.1
Span (Mb)	2,283,188,801
Number of contigs	168
Contig N50 length (Mb)	86,333,355
Number of scaffolds	147
Scaffold N50 length (Mb)	111,098,925
Longest scaffold (Mb)	250,357,659

**Table 2.** Chromosomal pseudomolecules in the genome assembly of *Tadarida brasiliensis*. ENA accession Chromosome Size (bp) GC%. The chromosome number of *Tadarida brasiliensis* is 2n= ***50***.

Genbank accession	Chromosome	Size (bp)	GC%
CM061257.1	1	250,357,659	41.0
CM061258.1	2	131,366,638	41.5
CM061259.1	3	123,954,994	43.2
CM061260.1	4	112,652,436	40.2
CM061261.1	5	112,580,834	42.5
CM061262.1	6	111,418,324	40.6
CM061263.1	7	111,368,843	42.0
CM061264.1	8	111,098,925	44.0
CM061265.1	9	107,746,758	40.7
CM061266.1	10	105,253,767	39.5
CM061267.1	11	101,937,717	40.8
CM061268.1	12	93,068,937	43.3
CM061269.1	13	90,219,467	42.9
CM061270.1	14	78,395,959	42.9
CM061271.1	15	70,705,328	43.6
CM061272.1	16	70,395,922	41.3
CM061273.1	17	66,982,268	46.3
CM061274.1	18	66,967,517	43.7
CM061275.1	19	53,271,390	44.8
CM061276.1	20	52,736,693	46.6
CM061277.1	21	38,494,270	43.7
CM061278.1	22	27,110,749	44.9
CM061279.1	23	15,501,133	47.4
CM061280.1	X	134,431,381	39.4
CM061281.1	Y	9,583,748	46.7

## Methods

The *T. brasiliensis* specimen was an adult male individual collected on the evening of October 15, 2018. The bat was caught by hand net as it left a roost located in a building on the Texas A&M campus in College Station, Brazos County, Texas, USA. Capture, handling, and sampling were approved by the local institutional Animal Care and Use Committee (Texas A&M University animal use protocol # 2017-0163D) and by Texas Parks and Wildlife scientific collecting permit SPR-1104-610.

Upon capture, a dichotomous key ( Schmidly, 2018) was utilized to confirm species identity. The bats were identified as *T. brasiliensis* based on specific morphological features such as forearm measures and other external features. Additionally, other molossids found in Texas ( *Eumops perotis*, *Nyctinomops femorosaccus*, *Nyctinomops macrotis*) are much larger compared to *T. brasiliensis*. At the capture location, there is limited range overlap with other molossids except for *N. macrotis* which is an uncommon species. However, these two species can be distinguished based on ear morphology as *N. macrotis’* ears meet on the midline of the head whereas *T. brasiliensis’* ears do not.

After collection from the field, the specimen was brought back to the laboratory and spent 5 months in captivity before tissue extraction and sample preparation. The animal was euthanized with pentobarbital overdose on February 28, 2019. Tissue samples collected were blood, brain (left hemisphere, cerebellum, front and back cortex, dorsal and ventral striatum), liver, heart (ventricle), left and right lung, spleen, left and right kidney, arm muscle, and left and right testicle. In total, 23 tissue samples were collected. All tissue samples were flash frozen in liquid nitrogen and stored in a -80°C freezer until shipment with the cold chain maintained. All data were recorded and reported in accordance with the ARRIVE guidelines ( Kilkenny *et al.*, 2010) – see data availability section and Table 1.

### Phenol-chloroform extraction of genomic DNA

Snap-frozen muscle tissue of *Tadarida brasiliensis* has been grinded into a fine powder in liquid nitrogen. Powdered muscle tissue was lysed overnight at 55°C in high-salt tissue lysis buffer (400 mM NaCl, 20 mM Tris base pH 8.0. 30 mM EDTA pH 8.0, 0,5% SDS, 100 ug/ml Proteinase K). RNA was removed by incubating in 50 mg/ml RNase A for 1 hour at 37°C. Cell debris have been removed by a centrifugation step. High molecular weight genomic DNA (HMW gDNA) was purified with two washes of Phenol-Chloroform-IAA equilibrated to pH 8.0, followed by two washes of Chloroform-IAA, and precipitated in ice-cold 100% Ethanol. HMW gDNA was collected by centrifugation, the gDNA pellet was washed twice in 70% cold Ethanol, dried for 10 min at 37°C, and eluted in 1x TE. Fragment length of the HMW gDNA was between 30 and 300 kb as shown by pulse field gel electrophoresis (PFGE) (Pippin Pulse, SAGE Science, Beverly, MA).

### Extraction of megabase-size gDNA

***a) Bionano-plug based megabase-size gDNA extraction for Bionano optical mapping.*** Megabase-size gDNA was extracted from liver tissue according to the Bionano Prep™ Animal tissue DNA isolation soft tissue protocol (Document number 30077, Bionano, San Diego, CA). In brief, liver tissue was homogenized in a tissue grinder directly followed by a mild Ethanol fixation. The homogenized tissue was embedded into agarose plugs and treated with Proteinase K and RNase A. Genomic DNA has been extracted from agarose plugs by agarose treatment and purified by drop dialysis against 1x TE. PFGE revealed megabase-size DNA molecule length of 50 kb up to 600 kb.

***b) Bionano-SP based megabase-size gDNA extraction for 10x linked Illumina reads.*** A second batch of megabase-size gDNA from snap-frozen kidney tissue was extracted with the beta version of the Bionano Prep SP Animal Tissue DNA Isolation Protocol (Document number 30339, Bionano, San Diego, CA). In brief, snap-frozen kidney tissue was homogenized with the Tissue Ruptor (Qiagen) on ice in a chaotropic buffer containing ethanol and tissue lysis took place by adding Proteinase K. Cell debris have been removed by centrifugation. The released gDNA was bound to a Nanobind disk (a novel nano structured silica on the outside of the thermoplastic paramagnetic disk) upon the addition of salting buffer and isopropanol. After several washing steps, the gDNA was eluted from the Nanobind disk. PFGE revealed mega-size DNA molecule length of 50 kb up to 600 kb.

### PacBio HiFi library preparation and sequencing

Three HiFi libraries of Phenol-chloroform extraction of genomic DNA (HMW gDNA) of *Tadarida brasiliensis* have been prepared as recommended by Pacific Biosciences according to the ‘Guidelines for preparing HiFi SMRTbell libraries using the SMRTbell Express Template Prep Kit 2.0 (PN 101-853-100, version 01). In summary, HMW gDNA has been sheared to 20 and 25 kb fragments, respectively, with the MegaRuptor™ device (Diagenode). 10 ug sheared gDNA have been used for library preparation. All PacBio SMRTbell™ libraries were size selected for fragments larger than 9 to 13 kb, 13 kb, and 15 kb with the BluePippin™ device according to the manufacturer’s instructions. The size selected libraries run on six Sequel II SMRT cells with the SEQUEL II sequencing kit 2.0 for 30 hours on the SEQUEL II of the DRESDEN concept Genome Center (DcGC), Germany. Circular consensus sequences were called making use of the default SMRTLink tools.

### Bionano optical mapping of megabase-size gDNA

Megabase-size gDNA of *Tadarida brasiliensis* was labelled as described in the Bionano Prep direct label and stain (DLS) protocol (Document number 30206). These DNAs were tagged with the nicking-free DLE enzyme. One flow cell of the labelled gDNA was run on the Bionano Saphyr instrument at the DcGC and circa 200X genome coverage of molecules longer than 150 kb was achieved.

### 10x linked reads

Linked Illumina reads were generated by using the 10x Genomics Chromium™ genome application following the Genome Reagent Kit Protocol v2 (Document CG00043, Rev B, 10x Genomics, Pleasonton, CA). In brief, 1 ng of mega-size genomic DNA was partitioned across over 1 Million Gel bead-in-emulsions (GEMS) using the Chromium™ devise. Individual gDNA molecules were amplified in these individual GEMS in an isothermal incubation using primers that contain a specific 16 bp 10x barcode and the Illumima ^®^ R1 sequence. After breaking the emulsions, pooled amplified barcoded fragments were purified, enriched, and went into Illumina sequencing library preparation as described in the protocol. Pooled Illumina libraries were sequenced to a 100X genome coverage on an Illumina NovaSeq instrument at the DKMS Life Science Lab gGmbH, Dresden, Germany.

### Hi-C chromatin confirmation capture

Chromatin confirmation capturing was done making use of the ARIMA-Hi-C (Material Nr. A510008) and the Hi-C+ Kit (Material Nr. A410110) and followed the user guide for animal tissues (ARIMA-Hi-C kit, Document A160132 v01 and ARIMA-Hi-C 2.0 kit Document Nr: A160162 v00). In brief, circa 50 mg flash-frozen powdered tissue was crosslinked chemically. The crosslinked genomic DNA was digested with the restriction enzyme cocktail consisting of two and four restriction enzymes, respectively. The 5’-overhangs are filled in and labelled with biotin. Spatially proximal digested DNA ends were ligated and finally the ligated biotin containing fragments were enriched and went for Illumina library preparation, which followed the ARIMA user guide for Library preparation using the Kapa Hyper Prep kit (ARIMA Document Part Number A160139 v00). The barcoded Hi-C libraries run on a NovaSeq6000 with 2x 150 cycles.

**Figure 3.**
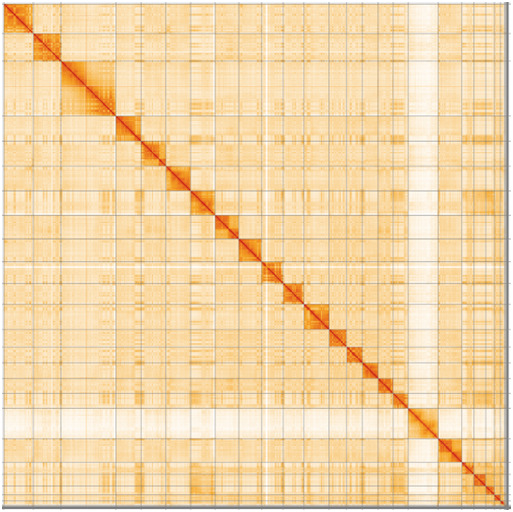
Hi-C Contact Map of the *T. brasiliensis* assembly with 25 chromosomes, visualized using HiGlass.

**Figure 4.**
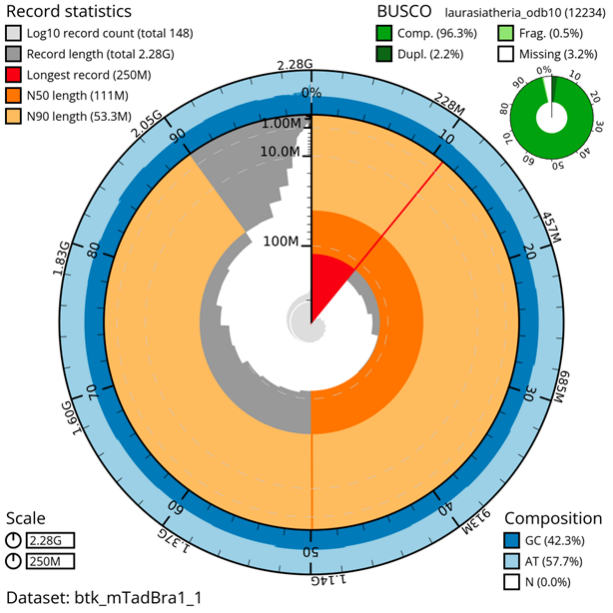
Genome assembly metrics generated using blobtoolkit for the *T. brasiliensis* genome assembly. The larger snail plot depicts scaffold statistics including N50 length (bright orange) and base composition (blue). The smaller plot shows BUSCO completeness in green.

**Figure 5.**
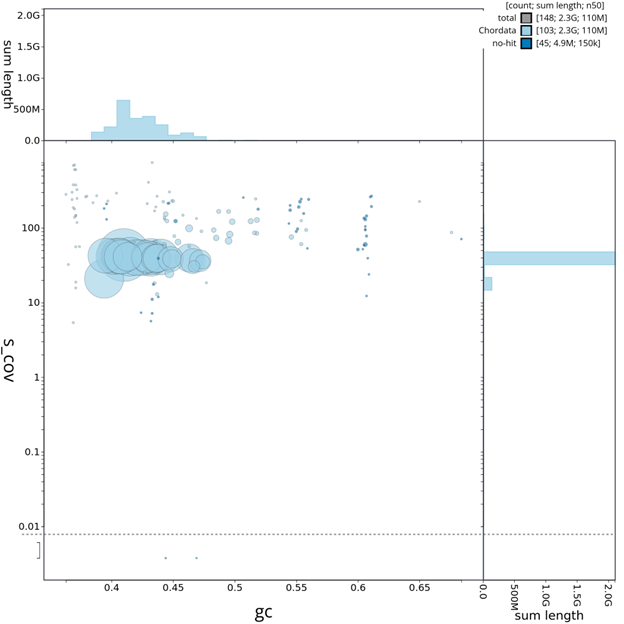
GC coverage plot generated for the *T. brasiliensis* assembly using blobtoolkit. Individual chromosomes and scaffolds are represented by each circle. The circles are sized in proportion to chromosome/scaffold length. Histograms show the sum length of chromosome/scaffold size along each axis. Color of circles indicate taxonomic hits of each Phylum represented in the assembly.

**Figure 6.**
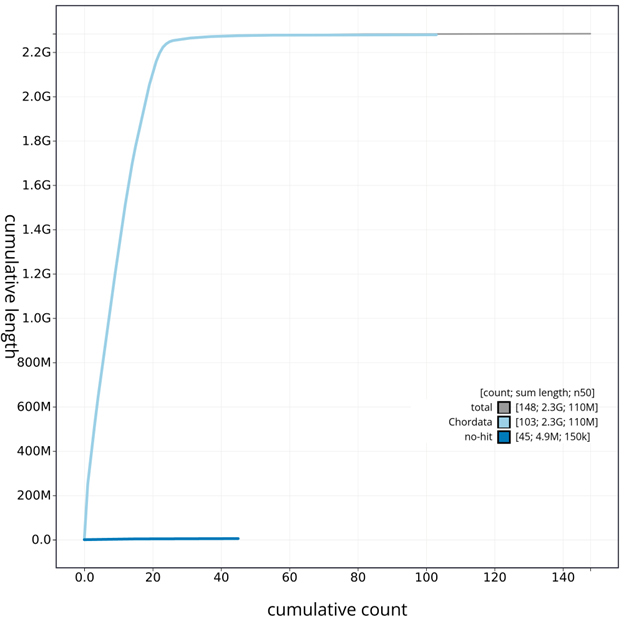
Cumulative sequence plot generated for the *T. brasiliensis* assembly using blobtoolkit. The grey line shows the cumulative length for all chromosomes/scaffolds in the assembly. Colored lines represent Phylum represented in the assembly.

Assembly was carried out following the Vertebrate Genome Project pipeline v2.0 ( Rhie *et al.*, 2020) as follows. HiFi reads were created with ccs (v6.0.0). HiFiasm (v0.16.0) was used to create the initial contig set. Haplotypic duplication was identified and removed with purge dups (v1.2.5) ( Guan *et al.*, 2020). The quality of the assembly was evaluated using Merqury ( Rhie *et al.*, 2020) and BUSCO ( Manni *et al.*, 2021). Scaffolding with 10X data was carried out with Scaff10X (commit bc3a0cb), Bionano data with Bionano Solve (v 3.6.1) and Hi-C data ( Rao *et al.*, 2014) with SALSA2 (commit e6e3c77) ( Ghurye *et al.*, 2019). HiGlass ( Kerpedjiev *et al.*, 2018) was implemented to generate Hi-C contact maps and perform manual curation of scaffolds into chromosomes. Figure 3, Figure 4, Figure 5 & Figure 6 were generated using BlobToolKit ( Challis *et al.*, 2020). Software utilized for *T. brasiliensis* analysis are depicted in Table 3.

**Table 3.** Software tools used.

Software tool	Version	Source
bamUtil	1.0.15	https://genome.sph.umich.edu/wiki/BamUtil:_bam2FastQ
FastQC	0.11.9	https://www.bioinformatics.babraham.ac.uk/projects/fastqc/
MultiQC	1.13	https://github.com/ewels/MultiQC
Genomescope	2.0	https://github.com/tbenavi1/genomescope2.0
HiFiasm	0.16.0	https://github.com/chhylp123/hifiasm
purge_dups	1.2.5	https://github.com/dfguan/purge_dups
BUSCO	5.3.2	https://busco.ezlab.org/
Merqury	1.3	https://github.com/marbl/merqury
Assembly-stats	17.02	https://github.com/rjchallis/assembly-stats
Scaff10X	4.2	https://github.com/wtsi-hpag/Scaff10X
Bionano Solve	3.6.1	https://bionano.com/software-downloads/
Arima-HiC Mapping Pipeline	-	https://github.com/ArimaGenomics/mapping_pipeline
SALSA	2.2	https://github.com/marbl/SALSA
HiGlass	1.11.7	https://github.com/higlass/higlass
samtools	1.9	https://www.htslib.org/
BlobToolKit	3.2.7	https://github.com/blobtoolkit/blobtoolkit

## Data Availability

The *T. brasiliensis* genome sequencing initiative is part of the Bat1K genome sequencing project. The genome assembly is released openly for reuse. The genome assembly can be found in the European Nucleotide Archive: *T. brasiliensis* (Brazilian free-tailed bat). Accession number: PRJNA972445, https://identifiers.org/ena.embl:PRJNA972445 ( Bat1K, 2023a). NCBI BioProject: *T. brasiliensis* isolate: mTadBra1 (Brazilian free-tailed bat). Accession number: PRJNA972445, https://www.ncbi.nlm.nih.gov/bioproject/PRJNA972445 under the Bat1K BioProject PRJNA489245 ( Bat1K, 2023b). This Whole Genome Shotgun project has been deposited in the DDBJ/ENA/GenBank repositories under the accession number GCA_030848815.1. The individual sequences are available here: https://www.ebi.ac.uk/ena/browser/view/GCA_030848815.1 ( Bat1K, 2023c). Data accession identifiers are reported in Table 1.
