# Supplementary material for: The genome sequence of Tadarida brasiliensis I. Geoffroy Saint-Hilaire, 1824 [Molossidae; Tadarida]
Source: Wellcome Open Res. 2024 Feb 29;9:98. [Version 1] doi: 10.12688/wellcomeopenres.20603.1 (PMC11128047; doi:10.12688/wellcomeopenres.20603.1)
Supplement: Peer review history

# Reviewer report: Reviewer response for version 1

**DOI:** 10.21956/wellcomeopenres.22806.r76586

**Published:** 2024-05-29

**Authors:** Lyre Murao

**Affiliations:** 1 University of the Philippines Mindanao, Tugbok District, Philippines

The first long-read genome assembly of *Tadarida brasiliensis *(Brazilian free-tailed bat) is reported in this work. Genomic studies will be beneficial for this species due to its unique traits, including as a mammalian model for the genetic and epigenetic basis of migration.  

 The sequencing methods and genome assembly are comprehensively outlined and appropriately designed based on the pipeline developed by the Vertebrate Genome Project of the Genome 10K Consortium. The combination of advanced sequencing technologies (HiFi, Hi-C, and Bionano) fittingly addressed the challenge of generating a high-quality haplotype genome assembly in the absence of a reference sequence. Post-assembly quality control revealed 96% of the genes have complete sequences.

 The results are clearly and comprehensively detailed. The sequence data is also made available, allowing further use for downstream analysis such as annotation, comparative genomics, and functional studies.

 I only have minor comments:

1. The short-read genome sequence of *T. brasiliensis* is already available. Although the authors argued on the importance of genomics research for this species, the need to generate a long-read genome assembly should be clearly communicated.
2. Does Figure 2 refer to the specific individual caught for this study or are these data obtained from other studies? While the expertise of the authors on bat species identification is unquestionable, actual data may be added to validate the claim on the bat’s identity, especially since it has close morphological features with other bats such as *N. macrotis*.
3. The sequencing coverage was 39X for HiFi and 100X for Bionano. How was the sequencing coverage decided? Was this estimated from the genome size of the organism?
4. The section Genome Sequence Report describes that the assembly obtained from HiFi long reads were scaffolded with Hi-C data. However, the role of Bionano genome mapping in the analysis was not clarified.
5. In the section Genome Sequence Report, it was claimed that 98.44%, of the assembly sequence was assigned to 25 chromosomal-level scaffolds. However, this statistic was not mentioned in the data section. How was this value obtained? Please clarify.
6. It would be worthwhile to mention in the main text that the alternative haplotype is also available.
7. The sequencing was appropriately performed for one individual bat, but it was noted that different tissues were used (muscle, kidney, liver) for each sequencing technology. While there may be a technical explanation for this, this should be communicated.
8. The data follow the format for similar manuscripts in this journal, however Figures 3-6 may not be understandable to a non-expert. Additional information in the figure caption or main text can help guide readers in the interpretation of data.
9. Were the authors able to make a comparison of the short-read and long-read genomes for *T. brasiliensis*?

Are sufficient details of methods and materials provided to allow replication by others?

Yes

Is the rationale for creating the dataset(s) clearly described?

Partly

Are the datasets clearly presented in a useable and accessible format?

Partly

Are the protocols appropriate and is the work technically sound?

Yes

Reviewer Expertise:

genomics, wildlife and disease spillover

I confirm that I have read this submission and believe that I have an appropriate level of expertise to confirm that it is of an acceptable scientific standard, however I have significant reservations, as outlined above.

# Reviewer report: Reviewer response for version 1

**DOI:** 10.21956/wellcomeopenres.22806.r76577

**Published:** 2024-05-25

**Authors:** Heliana Dundarova

**Affiliations:** 1 Institute of Biodiversity and Ecosystem Research, Bulgarian Academy of Sciences, Sofia, Bulgaria

The manuscript represents valuable research on the genome of the Brazilian free-tailed bat. 

 Approaches and methodologies are based on strong and well-documented protocols, as shown in the excellent implementation of results.

 The language of the manuscript is according to the good scientific practice. Such a report is valuable and will help future research on bat genomes.

 Minor comments:

 Keywords - please replace or delete *Tadarida brasiliensis *and genome sequence, because they are used in the title and can not be used as keywords. For example, use Brazilian free-tailed bat instead *Tadarida brasiliensis*.

 Species taxonomy - include also Vespertilionoidea, before Molossidae.

Are sufficient details of methods and materials provided to allow replication by others?

Yes

Is the rationale for creating the dataset(s) clearly described?

Yes

Are the datasets clearly presented in a useable and accessible format?

Yes

Are the protocols appropriate and is the work technically sound?

Yes

Reviewer Expertise:

Chiroptera, phylogeny, phylogeography, taxonomy

I confirm that I have read this submission and believe that I have an appropriate level of expertise to confirm that it is of an acceptable scientific standard.

# Reviewer report: Reviewer response for version 1

**DOI:** 10.21956/wellcomeopenres.22806.r76579

**Published:** 2024-05-25

**Authors:** Graham Etherington

**Affiliations:** 1 Earlham Institute, Norwich, UK

The authors report the first long-read assembly of the Mexican free-tailed bat *(Tadarida brasiliensis*). Bats are important models for a range of scientific study areas such as longevity, immunity, social structure, and migration. The authors provide an excellent introduction to this species as well as the rationale for generating a new assembly. I only have a few comments;

 1. The BioNano data does not appear to have been made publicly available and hence the report does not comply fully with Wellcome Open Research Data Policy. This should be uploaded to ENA as an 'Analysis' entry .https://ena-docs.readthedocs.io/en/latest/submit/analyses/bionano-maps.html

 2. In the methods section the authors mention they generate 10x Genomics and BioNano data, but there's no mention of using this in the Genome sequence report.

 3. In sentence three of the Introduction change, "While some genera with *Molossidae* .." to "While some genera within *Molossidae* ..."

Are sufficient details of methods and materials provided to allow replication by others?

Yes

Is the rationale for creating the dataset(s) clearly described?

Yes

Are the datasets clearly presented in a useable and accessible format?

Partly

Are the protocols appropriate and is the work technically sound?

Yes

Reviewer Expertise:

Genome assembly and conservation genomics.

I confirm that I have read this submission and believe that I have an appropriate level of expertise to confirm that it is of an acceptable scientific standard, however I have significant reservations, as outlined above.

# Reviewer report: Reviewer response for version 1

**DOI:** 10.21956/wellcomeopenres.22806.r76576

**Published:** 2024-04-18

**Authors:** Vincent J. Lynch

**Affiliations:** 1 Department of Biological Sciences, University at Buffalo, Buffalo, New York, USA

In this study, Webster et al. report the genome of *Tadarida brasiliensis.* I find that the rationale is well described and the methods are extensive, including Bionano, PacBio HiFi, 10x linked reads, and HiC. The genome quality looks excellent, and data are already available. I see no need for additional improvements or revisions to the manuscript.

Are sufficient details of methods and materials provided to allow replication by others?

Yes

Is the rationale for creating the dataset(s) clearly described?

Yes

Are the datasets clearly presented in a useable and accessible format?

Yes

Are the protocols appropriate and is the work technically sound?

Yes

Reviewer Expertise:

Evolutionary biology and genetics/genomics

I confirm that I have read this submission and believe that I have an appropriate level of expertise to confirm that it is of an acceptable scientific standard.
